# Tri-ponderal mass index as a screening tool for obesity prediction in children aged 6–9 years

**DOI:** 10.3389/fendo.2023.1277125

**Published:** 2023-11-09

**Authors:** Yang Niu, Yajie Zhang, Jinye Sheng, Wenyi Lu, Ji Li, Xiaomeng Mao, Wei Cai, Qingya Tang, Xiuhua Shen, Yi Feng

**Affiliations:** ^1^ Department of Clinical Nutrition, Xinhua Hospital Affiliated to Shanghai JiaoTong University School of Medicine, Shanghai, China; ^2^ Department of Clinical Nutrition, College of Health Science and Technology, Shanghai JiaoTong University School of Medicine, Shanghai, China; ^3^ Shanghai Institute for Pediatric Research, Shanghai, China; ^4^ Shanghai Key Laboratory of Pediatric Gastroenterology and Nutrition, Shanghai, China

**Keywords:** obesity, tri-ponderal mass index, body mass index, percentage of body fat, children

## Abstract

**Objective:**

This study aimed to evaluate the efficiency of tri-ponderal mass index (TMI) in determining obesity in Chinese children aged 6-9 years, using the criteria of percentage of body fat (PBF) and body mass index z-scores (BMI-Z).

**Methods:**

The cross-sectional study included 5365 children, aged 6–9 years, who participated in the project survey “Group prevention and treatment of obesity among students and school health promotion in Shanghai” from September 2007 to September 2009. Height, weight, waist circumference, body mass index (BMI), TMI, waist-to-height ratio (WHtR), and PBF were recorded. Statistical analyses including Kolmogorov–Smirnov test, chi-square test, receiver operating characteristics curve, and kappa chi-square test were performed.

**Results:**

TMI for both sexes was relatively constant with increasing age, and statistically significant differences were not observed at some ages (*P* > 0.05 at 6, 7, 8, and 9 years). WHtR showed subtle changes, while BMI and PBF increased significantly with age in boys and girls (*P* < 0.01). Using BMI-Z criteria as the measure of general obesity, the results indicated that TMI cutoff values for 6–9 years were 14.60 kg/m^3^ for boys and 14.84 kg/m^3^ for girls (*P* < 0.001). Analysis of the agreement between TMI and BMI-Z showed that the kappa statistic was 0.826 in boys and 0.709 in girls (*P* < 0.001).

**Conclusion:**

TMI, as a constant tool, holds great potential as an alternative screening method for identifying children aged 6-9 years who may be at risk of obesity at an early stage.

## Introduction

Obesity is a significant global public health concern, posing a serious threat to overall well-being. The World Health Organization (1) reports a sharp rise in the prevalence of overweight and obesity among children and adolescents (aged 5-19 years) from a mere 4% in 1975 to over 18% in 2016. In China, recent estimates reveal that 11.1% of children and adolescents aged 6-17 years are overweight, while 7.9% are with obesity, indicating a rapid increase over the past four decades (2).

Given rising prevalence of obesity among children and adolescents and the detrimental effects it can have, there is a pressing need for efficient and accessible screening methods to detect obesity early on. One commonly used indicator of obesity is the percentage of body fat (PBF) (3). However, due to the need for specialized equipment to measure PBF accurately, it is not practical for routine health care or for use in school-based settings (4).

Body mass index (BMI) and body mass index z scores (BMI-Z) are commonly used tools for screening obesity in children. However, unlike the standardized BMI thresholds for adults, BMI thresholds for childhood obesity vary by gender and age, making routine screening more challenging. It is worth noting that BMI is based on the assumption that adult weight is proportional to the square of height. However, in children and adolescents, the proportional relationship between weight and height should be around 3, not 2, due to the greater influence of height on weight during this stage of development. This can lead to instability in BMI measurements (5–9).

Tri-ponderal mass index (TMI) is a method for estimating body fat level that has been found to be more accurate than BMI, especially in non-Hispanic white adolescents aged 8–17 years. It is calculated by dividing weight (in kg) by height (in m^3^). TMI has also been shown to be superior to BMI-Z in classifying overweight status and discriminating central obesity in overweight adolescents (10). However, there is still uncertainty regarding whether TMI outperforms BMI or other adiposity indices in predicting obesity status in childhood, as indicated by a systematic review (11).

In order to address the limitations of previous studies, which showed inconsistent results and included a wide age range, our study aimed to evaluate the effectiveness of TMI compared to PBF and BMI-Z in determining obesity in a large sample of Chinese children aged 6-9 years. Additionally, we sought to develop sex-specific TMI cutoffs for screening obesity, based on the hypothesis that TMI, as a convenient indicator, would be stable and useful in children.

## Methods

### Participants and ethics approval

**Figure 1:**
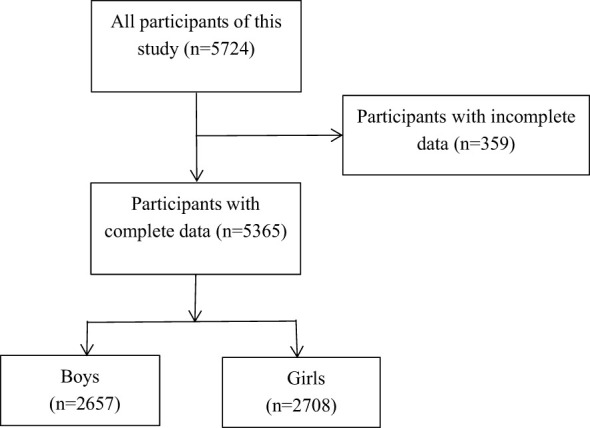
Flowchart of collecting data.

The cross-sectional study included 5365 healthy primary school students, aged 6–9 years, who participated in the project survey “Group prevention and treatment of obesity among students and school health promotion in Shanghai” from September 2007 to September 2009 (**Figure 1**). This study was approved by the Ethics Committee of Xinhua Hospital, School of Medicine, Shanghai JiaoTong University (No. XHEC-D-2023-173).

### Anthropometric assessments

Height, weight, and waist circumference (WC) were measured by trained health professionals at school. Height and weight were assessed with participants being in light clothing and with shoes removed, to an accuracy of 0.1 cm (height) and 0.1 kg (weight). WC was measured at the midpoint between the lower border of the rib cage and the iliac crest at the end of normal expiration with the arms naturally placed by the side, and recorded to the nearest 0.1 cm.

BMI, TMI, and waist-to-height ratio (WHtR) were calculated as follows: BMI (kg/m^2^) = (weight in kg)/(height in meters)^2^; TMI (kg/m^3^) = (weight in kg)/(height in meters)^3^; and WHtR = waist circumference (cm)/height (cm), respectively. PBF was evaluated using bioelectrical impedance analysis by whole-body impedance (TBF-410, Tanita, Japan). To accurately measure body mass percentage, participants should ensure they have an empty stomach and have not urinated for at least two hours prior to the measurement.

### Diagnosis of obesity

Age- and sex-specific BMI z-scores were estimated following the 2007 WHO Growth Reference Standards (12). BMI-Z is the most universally used and well-accepted screening indicator for obesity globally, and a deviation of ≥ 2 SD from the z-score of BMI-for-age is defined as obesity. The PBF threshold for obesity is 20% for boys aged 6–18 years, 25% for girls aged 6–14 years, and 30% for girls aged ≥ 15 years according to the “Guidelines on the prevention and control of overweight and obesity of school-age children and adolescents in China” (13, 14). Regarding abdominal obesity, a WHtR cutoff of ≥ 0.5 has been proposed and used as a threshold for both genders (15).

### Statistical analysis

Statistical analysis was performed using SPSS V.25.0 statistical software. Categorical variables were presented as frequencies and percentages. Quantitative variables were examined for normality of distribution using Kolmogorov–Smirnov test. Continuous variables were presented as the mean ± SD, whereas non-normally distributed data were presented as the median (p25, p75).

Stat tests were conducted to assess the differences sex and age groups. The Chi-square (χ2) test was used for categorical variables, while nonparametric tests were employed for continuous variables. For each sex, receiver operating characteristic (ROC) analysis and optimal cutoff point analysis were conducted to determine the most effective BMI and TMI cutoffs for identifying obesity, using the obesity cutoff criteria of BMI-Z ≥ 2 SD, PBF ≥ 20% in boys and ≥ 25% in girls, and WHtR ≥ 0.5. In addition, the kappa chi-square test was employed to assess the agreement between TMI and BMI-Z and PBF, thereby increasing confidence in the reliability of the measuring tool. All P values were calculated using two-sided tests, with a significance level of P < 0.05 set for each test.

## Results

### Characteristics of study participants

As shown in **Table 1**, a total of 5365 participants with a mean age of 8.4 (7.8, 8.9) years were recruited in this study. There were 2657 (49.52%) boys and 2708 (50.48%) girls. Boys had higher height, weight, WC, WHtR, PBF, fat mass (FM), TMI, and BMI than girls (all *P* < 0.001). There was no statistically significant difference in age between boys and girls (*P* > 0.05).

**Table 1:** Characteristics of the participants.

n	Total sample	Boys	Girls	*P* value
	5365	2657	2708	
Age, years	8.42 (7.75, 8.92)	8.42 (7.75, 8.96)	8.42 (7.75, 8.92)	0.432
Height, cm	132 (127, 137)	132.50 (127.70, 137.70)	131.30 (126.30, 136.37)	<0.001
Weight, kg	29.30 (25.20, 34.90)	30.60 (26.10, 36.75)	28.10 (24.40, 33.20)	<0.001
WC, cm	59 (54, 66)	60.50 (55, 69)	57.15 (53, 63.07)	<0.001
WHtR	0.45 (0.42, 0.49)	0.46 (0.43, 0.51)	0.44 (0.41, 0.47)	<0.001
PBF, %	17.70 (13.90, 23)	19.10 (15.15, 25)	16.40 (12.90, 21.20)	<0.001
FM, kg	5.13 (3.57, 7.93)	5.77 (4.04, 8.90)	4.58 (3.20, 6.93)	<0.001
TMI, kg/m^3^	12.80 (11.75, 14.36)	13.20 (12.04, 14.97)	12.50 (11.52, 13.77)	<0.001
BMI, kg/m^2^	16.77 (15.26, 19.09)	17.37 (15.66, 20.02)	16.29 (14.92, 18.31)	<0.001
Obesity				
BMI-Z ≥ 2, n (%)	889 (16.57)	663 (24.95)	889 (8.30)	<0.001
PBF ≥ 20%, n (%)	1570 (29.26)	1206 (45.38)	364 (13.44)	<0.001
WHtR ≥ 0.5, n (%)	1307 (24.36)	849 (31.95)	458 (16.91)	<0.001

The prevalence of central obesity according to WHtR ≥ 0.5 was 24.36% in the total sample, 25.3% in boys, and 12.2% in girls.

The prevalence of obesity defined as BMI-Z ≥ 2 SD was 24.95% in boys and 8.30% in girls (*P* < 0.001), while the prevalence of obesity defined as PBF ≥ 20% in boys and ≥ 25% in girls was 45.38% and 13.44%, respectively (both *P* < 0.001, **Table 1**).

### Obesity indices in different age groups

**Figure 2:**
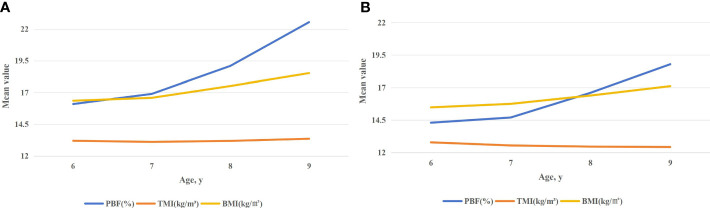
**(A)** Median (p25, p75) values of TMI, BMI, and PBF in boys aged 6–9 years. **(B)** Median (p25, p75) values of TMI, BMI, and PBF in girls aged 6–9 years.

TMI for both sexes was relatively constant with increasing age, and statistically significant differences were not observed at some ages (*P* > 0.05 at 6, 7, 8, and 9 years; **Table 2** and **Figures 2A, B**). Overall, WHtR showed subtle changes, while BMI and PBF increased significantly with age in boys and girls (*P* < 0.01; **Table 2** and **Figures 2A, B**).

**Table 2:** Median (p25, p75) values of TMI, BMI, WHtR, and PBF for age group 6–9 years.

Age, years	6	7	8	9	*P* value
Boys					
n	195	745	1157	664	
TMI, kg/m^3^	13.20 (12.24, 14.36)	13.11 (12.11, 14.68)	13.19 (11.96, 14.94)	13.36 (12.05, 15.40)	0.584
BMI, kg/m^2^	16.36 (14.92, 17.66)	16.59 (15.25, 18.75)	17.52 (15.70, 20.04)	18.53 (16.37, 21.47)	<0.001
WHtR	0.45 (0.42, 0.48)	0.45 (0.42, 0.50)	0.46 (0.43, 0.51)	0.47 (0.43, 0.53)	<0.001
PBF, %	16.10 (13.70, 21)	16.90 (14.10, 21.10)	19.10 (15.10, 25)	22.55 (17.70, 28.57)	<0.001
Girls					
N	115	758	1173	662	
TMI, kg/m^3^	12.79 (12.01, 13.82)	12.55 (11.61, 13.75)	12.46 (11.50, 13.78)	12.43 (11.38, 13.78)	0.062
BMI, kg/m^2^	15.48 (14.61, 16.93)	15.75 (14.54, 17.45)	16.39 (14.93, 18.35)	17.11 (15.55, 19.14)	<0.001
WHtR	0.43 (0.41, 0.45)	0.43 (0.41, 0.47)	0.44 (0.41, 0.48)	0.44 (0.41, 0.48)	0.02
PBF, %	14.30 (11.80, 17.80)	14.70 (12.10, 18.70)	16.60 (12.80, 21.20)	18.80 (14.70, 23.82)	<0.001

### Optimal TMI cutoff values in screening of obesity and central obesity

The sex- and age-based ROC curves for all of the obesity indices employed as predicting tools for the detection of pediatric general and central obesity are represented in **Table 3**. Using the BMI-Z criterion as the measure of general obesity, the results indicated that TMI cutoff values for 6–9 years were 14.60 kg/m^3^ (sensitivity 0.95, specificity 0.93) for boys and 14.84 kg/m^3^ (sensitivity 0.98, specificity 0.94) for girls (both *P* < 0.001, **Table 3**). When PBF was used as a criterion for general obesity, the TMI cutoff values for 6–9 years were 13.34 kg/m^3^ (sensitivity 0.80, specificity 0.79) for boys and 13.71 kg/m^3^ (sensitivity 0.96, specificity 0.84) for girls (both *P* < 0.001, **Table 3**). When WHtR served as a reference standard for central obesity, the TMI cutoff values for 6–9 years were 14.12 kg/m^3^ (sensitivity 0.91, specificity 0.91) for boys and 13.72 kg/m^3^ (sensitivity 0.91, specificity 0.87) for girls (both *P* < 0.001, **Table 3**).

**Table 3:** ROC curve analysis by BMI-Z ≥ 2SD, PBF (≥ 20% in boys, ≥ 25% in girls), WhtR ≥ 0.50 for TMI indice as predictor of obesity and central obesity by sex and age.

	BMI-Z ≥ 2SD					PBF (≥ 20% in boys, ≥ 25% in girls)					WhtR ≥ 0.50				
Age, y	6 - 9	6	7	8	9	6 - 9	6	7	8	9	6 - 9	6	7	8	9
Boys															
AUC	0.988	0.985	0.990	0.986	0.989	0.874	0.892	0.913	0.887	0.876	0.966	0.927	0.966	0.970	0.967
Cutoff	14.60	14.94	14.61	14.41	14.70	13.34	13.31	14.14	13.48	13.46	14.12	14.47	14.19	14.12	14.03
Sensitivity	0.95	0.93	0.95	0.97	0.95	0.80	0.92	0.79	0.80	0.71	0.91	0.84	0.92	0.92	0.90
Specifity	0.93	0.94	0.93	0.90	0.94	0.79	0.73	0.89	0.83	0.90	0.91	0.93	0.89	0.92	0.91
Youden	0.88	0.88	0.89	0.88	0.89	0.60	0.65	0.68	0.63	0.62	0.82	0.77	0.81	0.85	0.81
Girls															
AUC	0.993	1.000	0.997	0.991	0.991	0.966	1.000	0.978	0.970	0.977	0.957	0.988	0.954	0.949	0.974
Cutoff	14.84	15.84	14.98	15.05	14.81	13.71	15.84	14.98	14.10	13.65	13.72	14.52	13.72	13.71	13.36
Sensitivity	0.98	1.00	1.00	0.96	0.96	0.96	1.00	0.92	0.94	0.95	0.91	1.00	0.95	0.90	0.97
Specifity	0.94	1.00	0.96	0.96	0.94	0.84	0.99	0.96	0.91	0.90	0.87	0.94	0.86	0.87	0.85
Youden	0.92	1.00	0.96	0.92	0.91	0.81	0.99	0.88	0.86	0.85	0.79	0.94	0.81	0.77	0.83

### Kappa test among TMI, PBF, and BMI-Z

With respect to predicting obesity, ROC analysis showed that TMI ≥ 14.60 kg/m^3^ in boys and ≥ 14.84 kg/m^3^ in girls had a higher area under the curve (AUC), sensitivity, specificity, and Youden index than TMI ≥ 13.34 kg/m^3^ in boys and ≥ 13.71 kg/m^3^ in girls in both sexes in the age group from 6 to 9 years. Kappa chi-square test was further conducted among TMI (≥ 14.60 kg/m^3^ in boys and ≥14.84 kg/m^3^ in girls), BMI-Z (≥ 2 SD), and PBF (≥ 20% in boys and ≥ 25% in girls). The comparison between TMI and BMI-Z showed an agreement statistic kappa of 0.826 in boys and 0.709 in girls, which is considered good overall agreement (both *P* < 0.001, **Table 4**). The kappa values were 0.554 in boys and 0.719 in girls for the comparison between TMI and PBF, and 0.518 in boys and 0.676 in girls for the comparison between BMI-Z and PBF (both *P* < 0.001, **Table 4**).

**Table 4:** Consistency test between TMI, PBF and BMI-Z in boys and girls.

	TMI (≥ 14.60 kg/m³ in boys, ≥ 14.84 kg/m³ in girls)		BMI-Z ≥ 2SD	
	Kappa	*P* value	Kappa	*P* value
Boys				
PBF ≥ 20%	0.554	< 0.001	0.518	< 0.001
BMI-Z ≥ 2SD	0.826	< 0.001	–	–
Girls				
PBF ≥ 25%	0.719	< 0.001	0.676	< 0.001
BMI-Z ≥ 2SD	0.709	< 0.001	–	–

## Discussion

In this study, we assessed the reliability of TMI as an indicator for screening obesity in Chinese children. The findings indicated that TMI exhibited greater stability and consistency across different gender and age groups, in comparison to PBF and BMI. Consequently, TMI can be regarded as a more effective predictor of both general and central obesity. This conclusion is supported by the high AUC values (ranging from 0.874 to 1.000) observed in boys and girls of all age groups. Additionally, the high kappa values (0.826 in boys and 0.709 in girls) demonstrated excellent agreement, further strengthening the validity of TMI as an obesity-screening tool.

According to Peterson CM’s study (5) in children aged 8 to 17 years, TMI is positively associated with PBF and estimates PBF in children and adolescents more accurately than BMI does. In the present study, we found that PBF increased significantly with age, but the value of TMI remained stable, which is in line with the previous study (5). Furthermore, in pediatric Type 2 Diabetes patients (age 10.2-17.9 years), Haifa Alfaraidi (16) found that TMI is associated with adiposity, and even the components of the metabolic syndrome.

By using PBF as the reference standard for obesity, we obtained the cutoff values of TMI with higher AUCs (0.87 in boys and 0.99 in girls), which were closer to Antonino’s study (1.00 in boys and 0.99 in girls) with 35 children aged 8-9 years (17). However, considering the current judgment of childhood obesity, there is still a lack of unified reference standards for children’s PBF, and the TMI reference value of children with obesity obtained at present needs further discussion.

BMI-Z is a widely used tool for determining childhood obesity because it takes into account age and sex, and uses ≥ 2 SD (10, 12) as a uniform criterion for determining obesity in children and adolescents aged 5–19 years. However, the BMI-Z value needs to take into account different BMI values in different age groups, which limits its wide application in schools and families. In addition, BMI is limited by age, and our study found that TMI was a more appropriate indicator because it was relatively stable across age groups, and had a strong correlation with BMI-Z (r= 0.95, *P <*0.001) in other study (18).

Based on these considerations, we performed ROC curve analysis using a combination of BMI-Z and TMI as the criteria for determining obesity, with BMI-Z ≥ 2 SD considered as the threshold. The objective was to assess the predictive value of TMI in obesity. The analysis revealed cutoff values of 14.60 kg/m^3^ for boys and 14.84 kg/m^3^ for girls between the ages of 6 and 9 years. Surprisingly, the consistency test results of TMI and BMI-Z demonstrated a high level of agreement, with kappa values of 0.826 for boys and 0.709 for girls. This finding indicates a strong overall consistency, which serves as a notable highlight of this study.

WC and WHtR are widely utilized measures for diagnosing central obesity in children and adolescents (19–22). Previous research has demonstrated a positive correlation between TMI and increased WC and WHtR in individuals aged 7 to 20 years (23, 24). Moreover, TMI has been shown to accurately identify central obesity, as indicated by WHtR, in both male and female children and adolescents aged 6 to 18 years, surpassing the accuracy of BMI (25). Importantly, this optimal cut-off point for identifying obesity during childhood and adolescence remains consistent across different age and gender groups, highlighting its reliability (25).

In this study, WHtR ≥ 0.5 was used as the diagnostic criterion for central obesity, and the cutoff values of TMI were 14.12 kg/m^3^ for boys and 13.72 kg/m^3^ for girls, which fluctuated less in different age groups and were similar to the results from the study of girls aged 9–11 years (25). However, it remains to be further discussed whether the prediction cutoff values can be applied in the clinical context. This is because WHtR is a better predictor of abnormal glycolipid metabolism and metabolic syndrome than TMI (26, 27). Therefore, a more suitable obesity-screening or prediction index should be selected for different purposes. In contrast, TMI is easy to calculate, has relatively stable thresholds for different age groups, and is more easily applicable for general public use.

Based on our current understanding, this study is the first of its kind to explore the predictive value of TMI in determining obesity in Chinese children aged 6-9 years, using PBF and BMI-Z as the reference standards for assessing obesity. It is worth noting that our participants are school-aged children, free of any exceptional diseases except for a portion being overweight or with obesity. Additionally, we conducted a kappa consistency test to analyze the agreement among these three obesity indicators. However, it is important to interpret the findings with caution due to the limitations in terms of age and race. It is worth noting that the reliability of the results would have been enhanced if the study had included a larger sample from multiple centers.

In conclusion, as a valid and constant tool, TMI may be used for the early identification of children aged 6-9 years who may be at risk of obesity at an early stage. This suggests that TMI could potentially serve as a promising alternative screening tool for obesity in various settings, including homes, schools, and primary health care facilities. Nevertheless, additional research is imperative to establish the optimal TMI cutoff value for adolescents and to explore the intricate relationship between TMI, muscle mass, and the development and progression of metabolic diseases.

## Data availability statement

The raw data supporting the conclusions of this article will be made available by the authors, without undue reservation.

## Ethics statement

The studies involving humans were approved by Ethics Committee of Xinhua Hospital, School of Medicine, Shanghai JiaoTong University. The studies were conducted in accordance with the local legislation and institutional requirements. Written informed consent for participation in this study was provided by the participants’ legal guardians/next of kin.

## Author contributions

YN: Conceptualization, Data curation, Formal Analysis, Investigation, Methodology, Supervision, Writing – original draft, Writing – review & editing. YZ: Formal Analysis, Writing – original draft, Writing – review & editing. JS: Data curation, Investigation, Writing – review & editing. WL: Methodology, Validation, Writing – review & editing. JL: Data curation, Investigation, Supervision, Writing – review & editing. XM: Data curation, Investigation, Methodology, Writing – review & editing. WC: Investigation, Methodology, Supervision, Writing – review & editing. QT: Data curation, Project administration, Resources, Validation, Visualization, Writing – review & editing. XS: Formal Analysis, Methodology, Project administration, Resources, Supervision, Validation, Writing – original draft, Writing – review & editing. YF: Data curation, Investigation, Methodology, Project administration, Resources, Supervision, Validation, Visualization, Writing – original draft, Writing – review & editing.
